# *ggkegg*: analysis and visualization of KEGG data utilizing the grammar of graphics

**DOI:** 10.1093/bioinformatics/btad622

**Published:** 2023-10-16

**Authors:** Noriaki Sato, Miho Uematsu, Kosuke Fujimoto, Satoshi Uematsu, Seiya Imoto

**Affiliations:** Division of Health Medical Intelligence, Human Genome Center, The Institute of Medical Science, The University of Tokyo, 4-6-1 Shirokanedai, Minato-ku, Tokyo 108-8639, Japan; Department of Immunology and Genomics, Graduate School of Medicine, Osaka Metropolitan University, Osaka 545-8585, Japan; Division of Metagenome Medicine, Human Genome Center, The Institute of Medical Science, The University of Tokyo, Tokyo 108-8639, Japan; Department of Immunology and Genomics, Graduate School of Medicine, Osaka Metropolitan University, Osaka 545-8585, Japan; Division of Metagenome Medicine, Human Genome Center, The Institute of Medical Science, The University of Tokyo, Tokyo 108-8639, Japan; Department of Immunology and Genomics, Graduate School of Medicine, Osaka Metropolitan University, Osaka 545-8585, Japan; Division of Metagenome Medicine, Human Genome Center, The Institute of Medical Science, The University of Tokyo, Tokyo 108-8639, Japan; Division of Health Medical Intelligence, Human Genome Center, The Institute of Medical Science, The University of Tokyo, 4-6-1 Shirokanedai, Minato-ku, Tokyo 108-8639, Japan

## Abstract

**Summary:**

The Kyoto Encyclopedia of Genes and Genomes (KEGG) database serves as a valuable systems biology resource and is widely utilized in diverse research fields. However, existing software does not allow flexible visualization and network analyses of the vast and complex KEGG data. We developed *ggkegg*, an R package that integrates KEGG information with *ggplot2* and *ggraph*. *ggkegg* enables enhanced visualization and network analyses of KEGG data. We demonstrate the utility of the package by providing examples of its application in single-cell, bulk transcriptome, and microbiome analyses. *ggkegg* may empower researchers to analyze complex biological networks and present their results effectively.

**Availability and implementation:**

The package and user documentation are available at: https://github.com/noriakis/ggkegg.

## 1 Introduction

In omics analysis, researchers obtain data on various biological entities, such as genes, enzymes, and compounds, with the primary objective of investigating their functional significance and behavior in living organisms. To aid in this analysis, Kyoto Encyclopedia of Genes and Genomes (KEGG) provides a comprehensive set of tools and databases specifically designed to facilitate the comprehension of systems biology (Kanehisa and Goto 2000). KEGG is widely utilized in diverse research fields, including for analyses of gene and protein functions, metabolic pathways, signal transduction, and molecular basis of diseases. It serves as a valuable resource for bioinformatics analyses, particularly of biological pathway information within gene lists (Wu *et al.* 2021).

The analysis of the vast and complex biological data encompassed within the KEGG database necessitates network-based analyses and customizable visualization. Such analyses are supported by native tools such as the KEGG mapper (Kanehisa *et al.* 2022). Furthermore, various packages, primarily R packages, have been developed to programmatically analyze and control visualization of this information. For example, the R package *graphite* acquires pathways from sources such as KEGG and Reactome, stores them in the graphNEL format, and provides an interface for topological analyses (Sales *et al.* 2012). *KEGGgraph* also downloads KEGG PATHWAY information and converts it into a format that can be effectively analyzed in R (Zhang and Wiemann 2009). In addition, *pathview* allows retrieval of KEGG Markup Language (KGML) and image files from KEGG, enabling the output of images that reflect various user-defined values on the graph. Employing *graphviz* or native KEGG images, it generates high-quality visualizations that are widely used in diverse research (Luo and Brouwer 2013).

These packages greatly enhance omics analyses by utilizing KEGG information; however, several limitations must be considered. Firstly, no existing package provides a convenient network-based visualization and analysis of essential KEGG components beyond pathways, such as modules and networks, or facilitates their integration with other KEGG information. Furthermore, although global and overview maps depicting interrelated metabolic pathways encompass various elements, such as reactions, compounds, and KEGG ORTHOLOGY (KO), no library has implemented functionality for programmatically visualizing these characteristics in detail, which is deemed crucial for analyses such as functional analysis of many orthologs identified in microbiome analysis. Moreover, integration with other packages for omics analyses is considered important and seamlessly integrating analysis results, such as inferred gene regulatory networks and multiple enrichment analysis results, and combining customized KEGG plots with other plots generated in the R environment prove challenging.

Therefore, we developed *ggkegg* to extend these packages. *ggkegg* retrieves information such as the KEGG PATHWAY and MODULE, formats them into a structure that is easy to analyze, and offers a series of functions for further analyses and visualization. *ggkegg* can also be viewed as an extension of *ggplot2*, an R package that composes plots using the grammar of graphics (Wickham 2016) and serves as the foundation for visualization in numerous publications on bioinformatics and life sciences. In this paper, we present the R package *ggkegg* along with its characteristics, delineating them by functional categories and providing illustrative examples of their utilization in bioinformatics analyses.

## 2 Implementation

### 2.1 Parsing functions

One fundamental feature of the *ggkegg* package involves parsing, where the latest KEGG information is retrieved from the KEGG API, cached, processed, and output as a network in *tbl_graph* format in *tidygraph*, which is the format for tidy manipulation of networks, useful for network analyses (Pedersen 2023, https://github.com/thomasp85/tidygraph). KEGG information can be manipulated and analyzed using a grammar of data manipulation, *dplyr*, and directly visualized using the graph visualization library, *ggraph* (Pedersen 2022, https://github.com/thomasp85/ggraph). All the information in KGML is accessible in one *tbl_graph* object by taking additional steps such as parsing of group nodes like compounds by adding additional identifiers representing the groups, along with components belonging to these groups and edges indicating their relationships. Building on this, the calculation functions of various edge and node importance measures, such as the centrality degree found in *igraph* and *tidygraph* (Csárdi and Nepusz 2006), can be applied to KEGG graphs, and users can visualize the information in native ways, such as by changing the node size to a degree.

Furthermore, the parsing of crucial components other than the pathway, the KEGG MODULE, and the KEGG NETWORK into the same network format is supported. The KEGG MODULE is a set of functional units of genes and reactions, whereas the KEGG NETWORK depicts the molecular background of drugs and diseases through directed relationships. This enables analyses of modules and networks along with the pathway, allowing for a more detailed investigation of the functions of the identified genes and other components. These modules are composed of block units that can be parsed as text-based or graphical representations.

### 2.2 Plotting functions

Effective visualization is crucial for complex network data. This package aims to visualize KEGG information using the grammar of graphics, which constructs plots by describing the components of graphics. This can simplify complex visualization and is considered useful for analyses of KEGG data containing diverse elements, such as genes, compounds, and reactions.

We developed several functions within our package to enhance the visualization of KEGG elements. These functions expand the capabilities of *ggraph*, enabling the addition of rectangular nodes and simplifying the visualization of grouped nodes and edges. Moreover, our package allows for projection of external networks, such as gene regulatory networks, onto the KEGG map, facilitating comparison with networks inferred from other datasets. In addition, we enable the projection of R-generated *ggraph* onto original KEGG PATHWAY images, providing a wide range of visual expressions for representing analysis outcomes. This integration allows for application of various layers from *ggplot2* directly to native KEGG images. Furthermore, when nodes of interest span multiple pathways, we offer the flexibility to arrange native pathways in panels or use custom graph layouts, facilitating analyses of inter-pathway nodes and their roles.

The package also allows visualization of global and overview metabolism maps as graphs, incorporating information on KOs, compounds, and reactions associated with overall metabolism. In addition to showing normal pathways, these maps can be colorized based on statistical values derived from the analysis and functional classes of the compounds and reactions, allowing for an insightful overview of metabolic capacity. This feature proves particularly useful when utilizing ortholog information obtained from microbiome analyses. Furthermore, the package provides functions for annotating each node, like whether the nodes are included in the KEGG MODULE, and subsequently visualizing this annotated information.

For useful visualization of nodes ranging from compounds to genes from various organisms, it is possible to convert KEGG identifiers attached to the nodes in the graph into other identifiers (e.g. gene symbols, enzymes, and compound names) based on detailed criteria using the KEGG API.

### 2.3 Functions to work with other packages

For convenient integration with various packages for omics analyses in the R and Bioconductor environments, we prepared several functions. As a basic function, the integration of various numerical values for nodes and edges, such as expression matrices obtained from omics analysis or statistical values, into the *tbl_graph* object is supported. This simplifies graph-based analyses such as calculating edge betweenness using KEGG information and these values.

For example, *DESeq2* is a package widely used for differential expression or abundance analyses in transcriptomic and microbiome studies. The package allows direct incorporation of result tables, such as log2 fold change and *P*-values, into plots, thereby facilitating a more straightforward and profound comprehension of the analysis outcomes (Love *et al.* 2014). In addition, functions are provided to render the results of gene set enrichment analyses, commonly performed using packages such as *clusterProfiler*, onto pathways directly from the stored result (Wu *et al.* 2021).

### 2.4 Analysis functions

In addition to its visualization capabilities, *ggkegg* offers several functions for calculating module completeness, module abundance, and pathway abundance based on input KO data. Module completeness is assessed for each block in the module using logical expressions provided by KEGG. Module abundance is then determined by user-defined calculation based on the KO abundances and the module completeness. Similarly, pathway abundance can be summarized by the calculation such as mean or the sum of the modules within the pathway. This allows for not only assessment of KO presence but also comparison of module and pathway presence across different groups.

## 3 Results

### 3.1 Application in transcriptomic analysis

We present several examples of the analyses performed using *ggkegg*. The summary of functions implemented in *ggkegg* is described in Supplementary Fig. S1, and the links to the sample codes for reproducing figures are summarized in Supplementary Table S1. The detailed description of datasets used in the applications is available in Supplementary Text S1. First, we demonstrate the incorporation of multiple enrichment analysis results into KEGG information obtained from multiple datasets using the package. We used transcriptomic datasets to investigate transcriptomic changes in cells induced by infection with the BK polyomavirus (BKPyV). The BKPyV is a DNA virus that causes diseases in multiple organs of patients receiving immunosuppressive regimens after solid organ transplantation. Two datasets on infection in renal proximal tubular epithelial cells and normal urothelial cells (Assetta *et al.* 2016, Baker *et al.* 2022) were downloaded and processed using the *nf-core* pipeline (Ewels *et al.* 2020), and differentially expressed genes were identified using *DESeq2* (Love *et al.* 2014). Enrichment analysis was performed employing the *clusterProfiler* function *enrichKEGG()*, utilizing the genes upregulated after infection as input. We obtained multiple enriched pathways, Homologous recombination, Cell Cycle, and Fanconi anemia pathway in both datasets and joined pathway networks (Wu *et al.* 2021).

**Figure 1.**
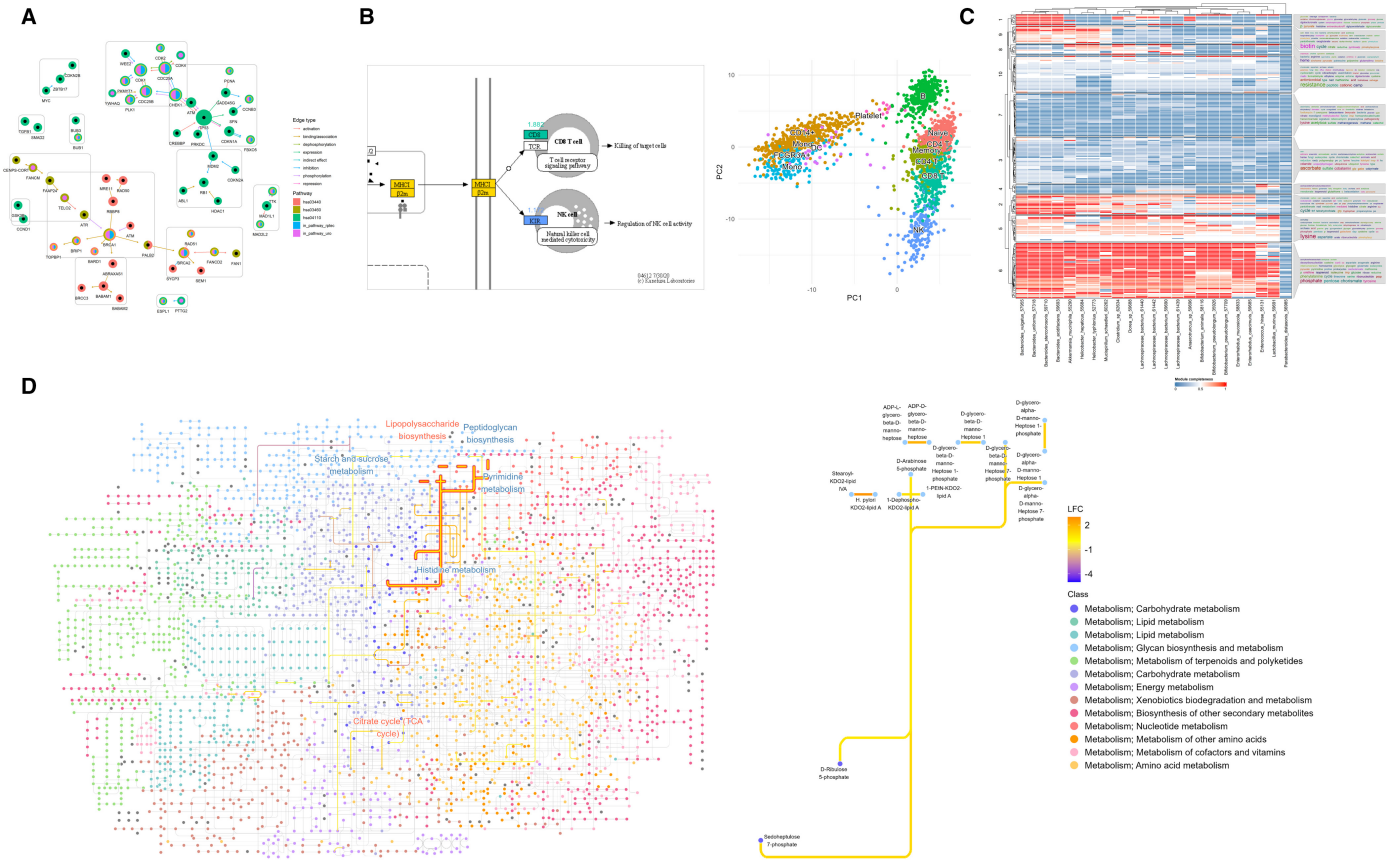
Summary of *ggkegg* utility. (A) Differentially expressed genes from two different datasets on cellular responses of BK polyomavirus infection in kidney and urothelial cells were identified. Their enriched pathways are shown within a single figure using a scatterpie chart and multiple KEGG PATHWAY networks. The background pie charts represent pathways, and foreground represent in which dataset the genes were significantly differed. The rectangles indicate clusters based on the walktrap graph-based node clustering algorithm. (B) An example of visualizing marker genes for clusters identified in single-cell transcriptomic analysis, based on enrichment analysis of marker genes. The blue color corresponds to the annotation of natural killer cells, the green color represents CD8+ T-cells, and the gold color represents marker genes of both clusters. (C) The analysis of KEGG MODULE completeness based on species-level pangenome information. Microbial gene annotations were obtained for enzyme commission numbers. The numbers were converted to KEGG ORTHOLOGY (KO), and module completeness was computed and visualized by a heatmap. Cell indicates module completeness values, and the row module and the column species. K-means clustering was performed and wordclouds of module description are visualized in the right of the plot. (D) A visualization of log2 fold changes (LFC) of KO on a global metabolic map, identified in the gut metagenomic study of patients with Crohn’s disease. The significantly different KOs were shown, and the node color represents metabolic class and edge colors were based on the LFC of KOs, and the enrichment information of the KEGG PATHWAY is also shown with text, whereby blue represents less abundant and red represents more abundant. The highlighted edges are those related to lipopolysaccharide biosynthesis, and the detail of the pathway is shown in the right plot.

Figure 1A illustrates the resulting plot, where each node is sized by degree and colored according to whether it is enriched in datasets using scatterpie plots, a combination of scatter plots and pie charts, and each edge corresponding to the original KEGG edge attribute (Yu 2023, https://CRAN.R-project.org/package=scatterpie). The genes enriched in both datasets are colored in gold. Walktrap clustering was performed on the overall network, and the results are presented as rectangles covering nodes. The differences in gene expression between multiple datasets, relationships among genes and clusters identified in network analysis across multiple pathways are comprehensible in one plot. Also, a specific cluster, including genes such as *CDK1*, *CDC25A*, and *CDC25B* was upregulated in both cell lines, suggesting this cluster is globally disrupted in BKPyV infection.

### 3.2 Application in single-cell analysis

The next example demonstrates the interpretation of marker genes identified by comparing cell clusters in single-cell analyses and elucidating their functional significance in KEGG PATHWAY. We utilized a dataset of peripheral blood mononuclear cells available from 10x Genomics. The data was processed using *Seurat*, and the markers of each cluster were identified using the *Seurat* function *FindAllMarkers()* at a specified threshold (Hao *et al.* 2021). Subsequently, enriched pathways for the markers of the two clusters were identified using over-representation analyses by *enrichKEGG()* function in *clusterProfiler*. These markers within the pathway Antigen Processing and Presentation, as well as their average log2 fold changes, were plotted on the raw pathway image with the same color as the reduced dimension plot (Fig. 1B). Cluster annotation of CD8+ T cells and natural killer cells was in agreement with the highlighted KEGG PATHWAY information. The role of the genes in the pathway, their association with other clusters, and their statistical values can be readily understood from the resulting plot.

### 3.3 Application in microbiome analysis

We next performed a metabolic functional analysis of microbial species. We first show the analysis of functional module completeness for bacterial species genomes. We obtained mouse gut metagenomic data from a study that investigated colorectal cancer and cigarette smoking (Bai *et al.* 2022). The reads were processed with *fastp*, and MIDAS was employed to assess the coverage of microbial genomes. The pan-genome information for each interesting species was obtained, and centroid gene annotations for enzyme commission numbers were converted to KO identifiers (Nayfach *et al.* 2016). Module completeness was assessed across all modules using the identified KOs as inputs. The results are presented in Fig. 1C using the *ComplexHeatmap* and *simplifyEnrichment* packages (Gu *et al.* 2016, Gu and Hübschmann 2023). They indicate the differences in the metabolic capabilities of the identified species based on KOs assigned to the corresponding genes. Here, the module completeness is defined as the fraction of complete blocks in the module.

We finally present an example of analyzing publicly available KO information identified in a study that investigated the gut microbiome of Crohn’s disease patients using global metabolic map (He *et al.* 2017). We integrated the analysis results described in Supplementary Text S1 and mapped them onto the global metabolic pathway, ko01100. The edge colors correspond to the log2 fold changes, and the significantly altered pathways are labeled on the map (Fig. 1D). We additionally described detail of the enriched pathway in Crohn’s disease patients, lipopolysaccharide biosynthesis, to show which compounds the interesting KOs are catalyzing. In this way, we could inspect how interesting KOs, such as differentially abundant KOs between groups, and their functions were distributed across a range of metabolic pathways.

## 4 Conclusion

We presented an R package *ggkegg* and three examples of its application in analyzing transcriptomic and microbiome datasets. All these analyses can be performed exclusively within the R environment. KEGG is one of the most widely used biological pathway databases, and *ggkegg* can connect to network analysis and visualization libraries in the R environment such as *ggplot2*, *ggraph*, and *tidygraph*. This maximizes the benefits of the grammar of graphics, thereby facilitating efficient and comprehensible visualization of complex biological pathway information in KEGG.

One limitation of the package is that KEGG MODULE, NETWORK, and PATHWAY raw files sometimes have irregular formatting, which may result in incorrect parsing. In such cases, the package provides warnings. This package is expected to become an essential tool for enhancing the understanding of analyses conducted in R and Bioconductor. It can greatly contribute to various analyses, including single-cell, bulk RNA-Seq, and microbiome analyses, by integrating network analysis and visualization. Moreover, while developing other packages using *ggplot2*, *ggkegg* may enable convenient importing and plotting, providing valuable assistance for future package development. In addition, it can be easily combined with plots from other R packages, further assisting in the creation of publication figures with customizable elements harnessing the power of *ggplot2* in the R environment. The documentation is linked from the GitHub repository, detailing use cases accompanied by codes.

## Supplementary Material

btad622_Supplementary_Data
