# Supplementary material for: ggkegg: analysis and visualization of KEGG data utilizing the grammar of graphics
Source: Bioinformatics. 2023 Oct 16;39(10):btad622. doi: 10.1093/bioinformatics/btad622 (PMC10612400; doi:10.1093/bioinformatics/btad622)
Supplement: btad622_Supplementary_Data [file btad622_supplementary_data.docx]

**Supplementary Figure S1. The overview of the functions in *ggkegg***


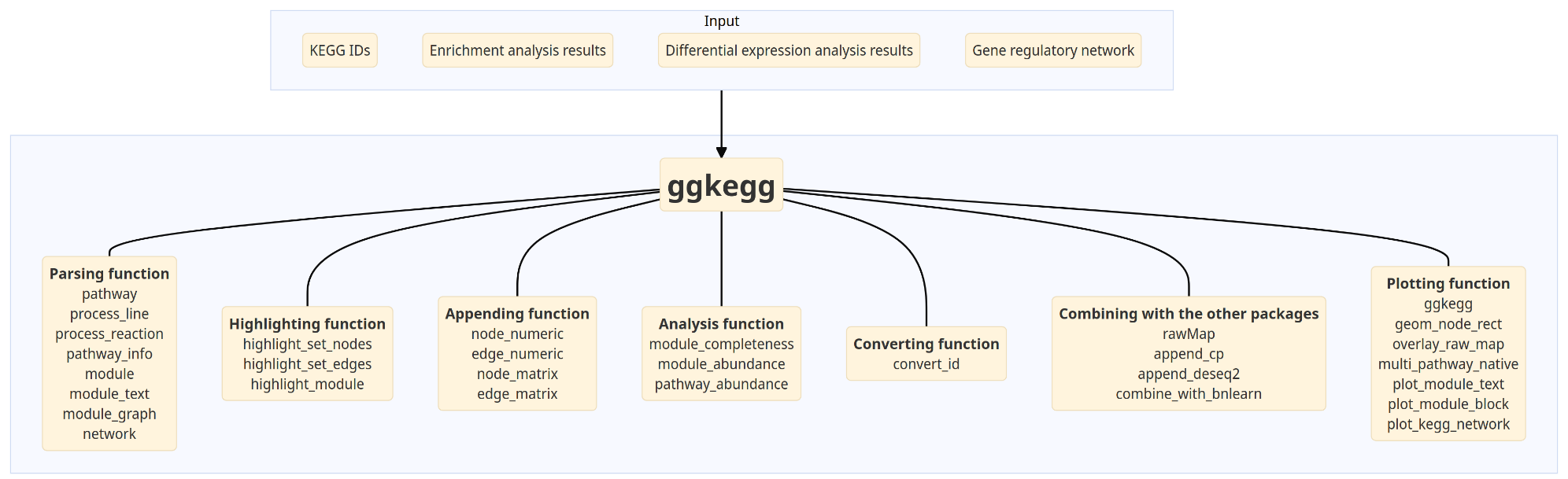


The figure is rendered by Mermaid.js.**Supplementary Table S1. The section in the documentation for producing the figures**

| **Figure** | **Section in the documentation** |
| --- | --- |
| Figure 1A | Usecases - Multiple enrichment analysis results across multiple pathways (https://noriakis.github.io/software/ggkegg/usecases.html) |
| Figure 1B | Usecases - Analyzing cluster marker genes in single-cell transcriptomics (https://noriakis.github.io/software/ggkegg/usecases.html) |
| Figure 1C | Module - Assessing module completeness across multiple microbial genomes  (https://noriakis.github.io/software/ggkegg/module.html) |
| Figure 1D | Pathway - Highlighting set of nodes and edges (https://noriakis.github.io/software/ggkegg/pathway.html) |

**Supplementary Text S1. The description of datasets used in the manuscript.**

**Figure 1A**

The raw FASTQ sequences of PRJNA560236 and PRJNA728925 were downloaded from Sequence Read Archive. The study depositing PRJNA560236 investigated time-series transcriptomic changes induced by BK and JC polyomavirus infection in renal proximal tubular epithelial cells, and PRJNA728925 investigated the transcriptomic changes induced by BK polyomavirus infection in the normal urothelial cells. The reads were processed with nf-core/rna-seq pipeline using Nextflow. *Salmon* was used to quantify the expression of transcripts, and the results were imported to perform differential expression analysis using *DESeq2*. The differentially expressed genes (DEGs) were identified at the significance threshold of false discovery rate-adjusted P-values below 0.05. The comparison was made between infected and control samples at 3 days post-infection for the renal proximal tubular epithelial cells in PRJNA560236, and between with and without viral infection for the normal urothelial cells in PRJNA728925. The DEGs were used as the input for *enrichKEGG()* function in *clusterProfiler*, and significantly enriched pathways were obtained. We selected Homologous recombination (hsa03440), Cell Cycle (hsa04110), and Fanconi anemia pathway (hsa03460) enriched in both datasets and merged pathway networks using node names, and appended the enrichment analysis results to the network. The walktrap node clustering algorithm was applied to the resulting network combining the pathways.

**Figure 1B**

The single-cell RNA-seq data of 3k peripheral blood mononuclear cells was obtained from the 10x Genomics website. The *Seurat* package was used to process dataset including normalization, scaling, principal component analysis, nearest-neighbor graph construction, and graph-based cell clustering. *FindAllMarkers()* function was used to identify the marker genes for the cell clusters. The identified marker genes were used as the input for *enrichKEGG()* function in *clusterProfiler*.

**Figure 1C**

The raw metagenomic reads were downloaded from Genome Sequence Archive at the National Genomics Data Center, Beijing Institute of Gemonics, Chinese Academy of Sciences/China National Center for Bioinformation under the accession number CRA006099. The reads were processed by *fastp*, and subsequently mapped to mouse reference genome (GRCm39) by Bowtie2. The unmapped reads were processed by MIDAS pipeline, using the default database as a reference. The profiled 23 species genome was used for the module completeness analysis.

**Figure 1D**

The KEGG ORTHOLOGY (KO) abundance data was downloaded from the GigaDB (dataset accession: 100317). The abundances were compared between the samples from Crohn’s disease and control by Welch’s t-test. The Bonferroni-corrected P-values of less than 0.01 were considered significant. Subsequently, over-representation analysis was performed on differentially abundant KOs using *MicrobiomeProfiler*. The significantly enriched pathways at the Bonferroni-corrected P-values of less than 0.01 were obtained. The functional classes for each HEX color value were inferred using *MicrobiomeProfiler* using the functional class of the pathway of the top hit for corresponding HEX values.
